# Molecular Characterization and Mutational Analysis of Clarithromycin- and Levofloxacin-Resistance Genes in *Helicobacter pylori* from Gastric Biopsies in Southern Croatia

**DOI:** 10.3390/ijms241914560

**Published:** 2023-09-26

**Authors:** Ivica Šamanić, Blanka Dadić, Željka Sanader Maršić, Mia Dželalija, Ana Maravić, Hrvoje Kalinić, Pavle Vrebalov Cindro, Željko Šundov, Marija Tonkić, Ante Tonkić, Jonatan Vuković

**Affiliations:** 1 Department of Biology, Faculty of Science, University of Split, Ruđera Boškovića 33, 21000 Split, Croatia; dadicblanka570@gmail.com (B.D.); mdzelalij@pmfst.hr (M.D.); amaravic@pmfst.hr (A.M.); 2 Department of Physics, Faculty of Science, University of Split, Ruđera Boškovića 33, 21000 Split, Croatia; zeljka.sanader@pmfst.hr; 3 Department of Compute Science, Faculty of Science, University of Split, Ruđera Boškovića 33, 21000 Split, Croatia; hkalinic@pmfst.hr; 4 Department of Gastroenterology, University Hospital of Split, 21000 Split, Croatia; pvrebalocindro@yahoo.com (P.V.C.); zsundov@gmail.com (Ž.Š.); ante.tonkic@mefst.hr (A.T.); 5 Department of Internal Medicine, School of Medicine, University of Split, 21000 Split, Croatia; 6 Department of Medical Microbiology and Parasitology, School of Medicine, University of Split, 21000 Split, Croatia; mtonkic@kbsplit.hr

**Keywords:** antibiotic resistance, *23S rRNA*, *gyrA*, *gyrB*, point mutations, molecular docking

## Abstract

Point mutations in the *23S rRNA*, *gyrA*, and *gyrB* genes can confer resistance to clarithromycin (CAM) and levofloxacin (LVX) by altering target sites or protein structure, thereby reducing the efficacy of standard antibiotics in the treatment of *Helicobacter pylori* infections. Considering the confirmed primary CAM and LVX resistance in *H. pylori* infected patients from southern Croatia, we performed a molecular genetic analysis of three target genes (*23S rRNA*, *gyrA,* and *gyrB*) by PCR and sequencing, together with computational molecular docking analysis. In the CAM-resistant isolates, the mutation sites in the *23S rRNA* gene were A2142C, A2142G, and A2143G. In addition, the mutations D91G and D91N in GyrA and N481E and R484K in GyrB were associated with resistance to LVX. Molecular docking analyses revealed that mutant *H. pylori* strains with resistance-related mutations exhibited a lower susceptibility to CAM and LVX compared with wild-type strains due to significant differences in non-covalent interactions (e.g., hydrogen bonds, ionic interactions) leading to destabilized antibiotic–protein binding, ultimately resulting in antibiotic resistance. Dual resistance to CAM and LVX was found, indicating the successful evolution of *H. pylori* resistance to unrelated antimicrobials and thus an increased risk to human health.

## 1. Introduction

*Helicobacter pylori* is a Gram-negative bacterium belonging to the class *Epsilonproteobacteria*, order *Campylobacterales*, family *Helicobacteraceae,* and genus *Helicobacter*. Today, more than 40 species belonging to the genus *Helicobacter* have been described and classified into species able to colonize the stomach (*H. pylori*) or the intestine (*H. cinaedi* and *H. fennelliae*) [1]. Humans are the main reservoir of *H. pylori*, and it is thought to be transmitted by oral or fecal–oral routes [2,3]. *H. pylori* infection is usually acquired in childhood and can be lifelong. *H. pylori* primarily colonizes the gastric mucosa but has also been found in the dental plaque and saliva of infected individuals [3]. Pathogenesis is associated with virulence factors that trigger inflammatory processes and alter and control host cellular responses. Virulence factors such as urease, VacA (vacuolizing toxin), CagA (cytotoxin), and lipopolysaccharides protect the bacterium, promote the colonization of the gastric mucosa, and prevent the host immune response, and thus may cause carcinogenesis.

The International Agency for Research on Cancer and the World Health Organization (WHO) have also classified *H. pylori* as a first category carcinogen because it is one of the most important etiologic factors for gastric cancer, which can be prevented by early eradication before metaplasia and atrophy occur [4].

*H. pylori* infection poses a clinical challenge as it is associated with gastritis, gastric and duodenal ulcers, MALT (mucosa-associated lymphoid tissue) lymphoma, and gastric cancer [5,6,7,8]. In addition, the eradication of *H. pylori* in high-risk areas reduces the risk of gastric cancer in asymptomatic individuals [9]. The Kyoto Consensus of 2015 defined *H. pylori* gastritis as an infectious disease and, importantly, in need of treatment, regardless of symptomatology [10]. The treatment of the infection requires a combination of antibiotics, antisecretory agents, and proton pump inhibitors [3].

Treatment usually begins with therapy consisting of clarithromycin (CAM) in combination with amoxicillin, metronidazole, and proton pump inhibitors administered for 14 days [11,12]. CAM is used as a “first line of defense” because of its effective action against infection. In cases where initial therapies are unsuccessful, secondary therapies such as levofloxacin (LVX) triple therapy (LVX, amoxicillin, and proton pump inhibitor) are used [11]. However, *H. pylori* antibiotic resistance is rising globally, and eradication therapy is failing, demanding a new treatment strategy [13,14]. Antibiotic resistance is a very large and important medical problem [15]. CAM resistance is above 15% worldwide except in Indonesia (9%), metronidazole resistance is 45% to 55% except in China (78%), and LVX resistance is 14% to 20% except in Indonesia (31%), with a propensity to rise with time and vary between regions [16]. 

In Europe, *H. pylori* resistance to CAM remained above 15% in naive patients in 2013–2020, and resistance to LVX (20%) and dual or triple resistance (13% and 6% of cases, respectively) were high [15]. A progressive decrease in metronidazole resistance rates was observed between the 2013–2016 (33%) and 2017–2020 (24%) periods [15].

Treatment adherence, patient behavior, and leftover antibiotics should be identified as potential targets for restraining antibiotic resistance [17]. The cause of resistance to CAM and LVX are point mutations in the genetic sequence that disrupt the cellular activity of antibiotics by altering the drug target site [18]. The *23S rRNA* mutations in the peptidyl transferase center (PTC) and adjacent regions V and VI can alter the binding affinity of CAM to the ribosome, thereby reducing its inhibitory effect on protein synthesis [19]. Point mutations in the quinolone resistance-determining regions (QRDRs) of the *gyrA* gene interfere with the binding of LVX to DNA gyrase, thereby impairing its ability to inhibit DNA replication and repair [20].

In Croatia, increasing primary antibiotic resistance to CAM and LVX has been observed in *H. pylori* strains [16]. The resistance rate among 40 strains isolated from January 2019 to January 2020 in southern Croatia was 37.5% (*n* = 15) to CAM and 5% (*n* = 2) to LVX [21].

As far as we know, the gene mutation conferring resistance to CAM and LVX has not been studied in Croatia thus far. Therefore, the aim of the study was the molecular characterization and structural modification analysis of three genes whose mutations are associated with resistance to the antibiotics CAM and LVX. The incorporation of computational molecular docking analysis provides insight into the atomic-level interactions between antibiotics and target proteins in wild-type bacteria and mutants with point mutations in the *23S rRNA*, *gyrA*, and *gyrB* genes and helps us to understand how these mutations affect the binding of antibiotics to their targets. To our knowledge, this is the first time that the GyrA and GyrB proteins have been used directly from *H. pylori* rather than as homologs for mutation analysis. In addition, resistance-related mutations were introduced into the protein structure and analyzed using computational techniques.

## 2. Results

### 2.1. Genetic Mutation Study

Mutations in genes related to antibiotic resistance play an important role in the mechanisms of antibiotic resistance; thus, we examined the mutational characteristics of the *23S rRNA*, *gyrA*, and *gyrB* genes of *H. pylori* in relation to resistance to the most commonly used antibiotics CAM and LVX. The genomic sequences of the *23S rRNA*, *gyrA*, and *gyrB* genes were PCR-amplified and sequenced.

#### 2.1.1. Nucleotide Sequence Analysis of the V Domain of the *23S rRNA* Gene of *H. pylori* Isolates and Molecular Docking

**Figure 1:**
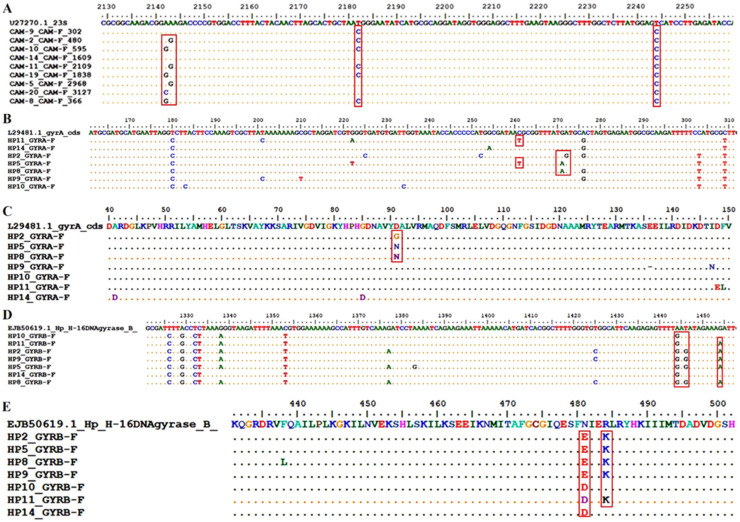
Multiple sequence alignment of *23S rDNA*, *gyrA*, and *gyrB* genes compared with reference genes. (**A**) *23S rRNA* nucleotide sequences of *H. pylori*. (**B**) gyrA nucleotide sequences of *H. pylori*. (**C**) GyrA amino acid sequences of *H. pylori*. (**D**) gyrB nucleotide sequences of *H. pylori*. (**E**) GyrB amino acid sequences of *H. pylori*. Mutation sites and amino acid changes are indicated in boxes.

The sequences of the V domain of the *23S rRNA* gene were compared with the reference sequence (GenBank U27270.1) (Figure 1A).

An analysis of the nucleotide sequences revealed mutations at positions 2142 and 2143, where the transition from adenine to guanine (A2142G and A2143G) and the transversion from adenine to cytosine (A2142C) occurs. The A2142G mutation was found in the isolates HP8, HP10, and HP19 (33.33%), and the A2142C mutation was found in the isolate HP20 (11.11%) (Table 1). The A2143G mutation was found in the isolates HP2, HP5, and HP11 (33.33%) (Table 1). Additional mutations were found at positions 2182 and 2244, where a transition from thymine to cytosine occurs (T2182C and T2244C). The T2182C mutation was found in the isolates HP2, HP8, HP9, HP10, HP11, and HP19 (66.67%), and the T2244C mutation was found in all isolates (Table 1).

**Figure 2:**
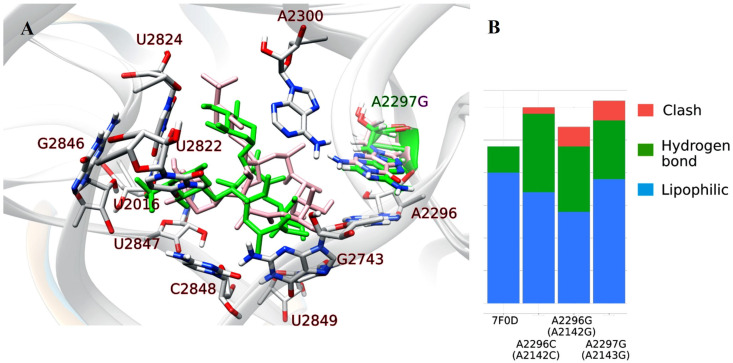
Summary of the molecular docking analyisis for 23S rRNA. (**A**) Left panel: Docked position of CAM on 23S rRNA (wild type and A2297G in M. tuberculosis mutant). (**B**) Right panel: Interaction analysis between CAM and different 23S rRNA mutants.

To better understand the influence of the mutations on antibiotic resistance, we investigated the structural properties of the 23S rRNA and CAM complex using molecular docking tools. The crystal structure of this complex from the pathogen *H. pylori* is lacking, but there is a cryo-EM structure of the *Mycobacterium tuberculosis* 50S ribosomal subunit bound with bound CAM [22]. Sequence comparison revealed that their 23S rRNA subunits share a 58% similarity and that the key nucleic bases mutated in this work are the same which allowed us to use *M. tuberculosis* 23S rRNA (PDB: 7F0D) for docking analysis. The numbering of the nucleic bases is different and to avoid confusion we have retained the numbering from *H. pylori* in the text but pointed out its numbering in *M. tuberculosis* as well. In Figure 2, the nucleic bases are from *M. tuberculosis* so its numbering is retained. A visual inspection of the *M. tuberculosis* 23S rRNA revealed that the T2182 and T2244 nucleic bases (corresponding to T2335 and T2397 in *M. tuberculosis*) are spatially separated from the CAM binding site and do not interact with CAM (see Figure S1). For this reason, these mutations were not further investigated. The docking procedure (described in the Section 4) was performed on the three mutants: A2296C, A2296G, A2297G. The best docked poses were selected and further analyzed to identify non-covalent interactions. The results are summarized in Figure 2, where the docked pose for the wild type and A2143G mutant are overlapped and hydrogen bonds, lipophilic interactions, and clashes are summarized.

#### 2.1.2. Nucleotide Sequence Analysis of the DNA Gyrase, Subunit A (*gyrA*) Gene from *H. pylori* Isolates and Molecular Docking

An analysis of the nucleotide sequences of the *gyrA* gene (Figure 1B) revealed a mutation at position 261 (C261T), where the transition from cytosine to thymine occurred. The mutation was found in the isolates HP5 and HP11 (28.57%). A G271A mutation (transition from guanine to adenine) was found at position 271 and was present in the isolates HP5 and HP8 (28.57%).

An A272G mutation (transition from adenine to guanine) is located at the next position, 272, and was found in the isolate HP2 (14.29%). An analysis of the amino acid sequences of DNA gyrase, subunit A (GyrA), revealed amino acid sequence changes as a result of missense mutations (Figure 1C).

According to the reference sequence of the *H. pylori gyrA* gene (GeneBank accession number L29481.1) (Figure 1B), the codon AAC is translated into the amino acid asparagine (N) (Figure 1C). In the sequences of the isolates HP5 and HP11, a change in the codon AAC to AAT was detected due to the C261T mutation (Table 2). Despite the mutation, the AAT sequence encodes the same amino acid, asparagine (N). Therefore, it can be concluded that the C261T mutation is a silent mutation.

In the reference sequence (Figure 2C), the triplet GAT encodes aspartic acid (D). In the HP5 and HP8 isolates, the G271A mutation results in a triplet switch from GAT to AAT, leading to a substitution of aspartic acid (D) for asparagine (N). This substitution is referred to as D91N because it was found at position 91 in the amino acid sequence (Table 2). In addition, the aforementioned triplet in the HP2 isolate was changed to GGT by the A272G mutation. The change in the triplet results in the replacement of aspartic acid (D) with glycine (G) and is referred to as D91G.

**Figure 3:**
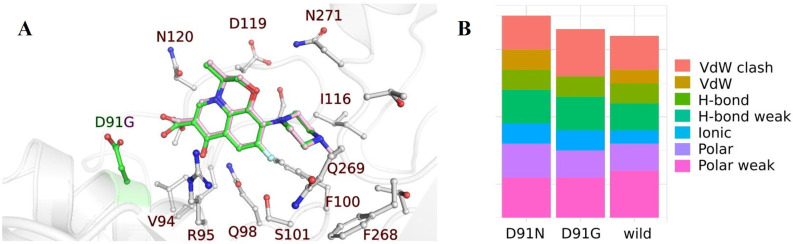
Summary of the molecular docking analysis for GyrA. (**A**) Docked poses for the wild type and D91G mutant of GyrA are shown in the left panel. Green-colored carbon atoms are part of the wild type structure, while the light pink color represents the mutated residue D91G and LVX docked within the mutant. The rest of the atoms are the same for the wild type and mutant and they are silver colored. (**B**) The number and types of the interactions are summarized in the right panel with the color legend, where VdW denotes for Van der Waals, H-bond denotes a hydrogen bond.

Additional analysis included the docking of LVX to GyrA in the wild type and two mutants (D91N and D91G). The chemical structure of LVX consists of several subunits which include a fluorinated quinolone core and a piperazinyl ring which make its structure very rigid with only five bonds open to torsions. This makes its docking relatively simple because spatial restrictions define its position and functional groups (methyl and carboxilic group) can be rotated to form non-covalent interactions. On the left panel of Figure 3, we present the structure of the wild type and D91G mutant with the docked LVX. The mutated amino acid D91 is highlighted in the green color (wild type), while the mutant with glycine has only a hydrogen in the side chain, which is not shown in the image. LVX atoms that belong to the docked position in the wild type are colored green, and in the D91G mutant, they are light pink. Other atoms in the structure of GyrA are the same, and they are shown in gray. It is evident that the docked poses of LVX are similar, which is an expected result of its rigid structure. On the right side, there is a summarized representation of all non-covalent interactions in the wild type and mutants. Interacting amino acid partners are emphasized in the left panel and include V94, R95, Q98, F100, S101, I116, D119, N120, F268, Q269, and N271. The number of polar interactions is similar in all variants, while the wild type has less destabilizing VdW clashing than the D91G mutant.

#### 2.1.3. Nucleotide Sequence Analysis of the DNA Gyrase, Subunit B (*gyrB*) Gene from *H. pylori* Isolates and Molecular Docking

Examination of the nucleotide sequences of the *gyrB* gene (Figure 1D) revealed the mutation A1444G at position 444, which was found in all isolates. The mutation T1446G was detected in the isolates HP2, HP5, HP8, and HP9 (57.14%). In addition, the mutation G1454A was observed at position 1454 and found in the isolates HP2, HP5, HP8, HP9, and HP11 (71.43%).

The observed DNA changes are missense mutations that result in different amino acids at a specific position (Figure 1E). For example, the codon AAT is translated to the amino acid asparagine (N), but in the isolates HP2, HP5, HP8, and HP9, the mutations A1444G and T1446G cause its conversion to the codon GAG (Table 3). The consequence of the described change is the replacement of asparagine (N) by glutamic acid (E). This substitution is referred to as N481E because it was found at position 481 in the amino acid sequence (Table 3). In addition, the isolates HP10, HP11, and HP14 contain the mutation A1441G, which replaces the same codon (AAT) with a new codon GAT. The newly formed codon is translated into aspartic acid (D), and the mutation is designated as N481D (Table 3). According to the reference sequence of the *H. pylori gyrB* gene (accession number EJB50619.1), the triplet AGA encodes the amino acid arginine. However, the mutation G1454A causes a change in the codon to AAA, replacing arginine (R) with lysine (K). The mutation is referred to as R484K (Table 3).

When the phenotypic susceptibility of *H. pylori* strains to LVX was examined, the isolates HP2, HP5, and HP8 were found to be resistant to the aforementioned antibiotic which is consistent with the results of bioinformatic analysis (Table 4).

**Figure 4:**
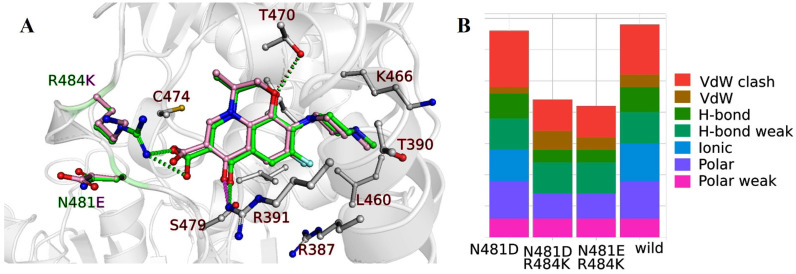
Summary of the molecular docking analysis for GyrB. (**A**) Docked poses for the wild type and N481E&R484K mutant of GyrB are shown in the left panel. Green-colored carbon atoms are part of the wild type structure, while the light pink color represents mutated residues (E481 and K484) and LVX docked with them. The rest of the atoms are the same for the wild type and the mutant and they are silver colored. (**B**) The number and types of the interactions are summarized in the right panel with the color legend.

Further investigations included the docking of LVX onto GyrB in the wild type, as well as three mutant forms (N481D, N481D&R484K, N481E&R484K). As already mentioned, the LVX chemical structure comprises distinct components which impart a significant rigidity. As a result, docking becomes relatively straightforward due to spatial constraints dictating its placement, while functional groups like methyl and carboxylic groups can be rotated to establish non-covalent interactions. This can be seen on the docking results summarized in Figure 4. On the left panel, the overlapped structure of the wild type and N481E&R484K mutant are presented with the docked LVX. The mutated amino acids N481 and R484 are colored green (wild type), while the mutant N481E&R484K atoms are light pink. Other gray-colored atoms in the structure of GyrB are the same for all variants. A condensed overview of all non-covalent interactions in the wild type and mutants is presented on the right-hand side, while the amino acid partners engaged in interactions are highlighted in the left panel, encompassing R387, T390, R391, L460, K466, T470, C474, and S479. The number of ionic interactions changes depending on the mutation where the amino acid with charged side chain (N) is replaced with a polar non charged residue (D, E), resulting in the fewer interactions.

## 3. Discussion

In approximately 50% of the world’s population, *H. pylori* colonizes the gastric mucosa and causes various gastrointestinal diseases, but it does not cause symptoms in a large proportion of infected individuals [6,18]. Treatment is particularly challenging, mainly because monotherapies do not show success, but according to the EHSG (European Helicobacter Study Group) guidelines, a combination of antibiotics (CAM, amoxicillin, metronidazole, LVX, etc.) with proton pump inhibitors is required [7]. Despite the use of combination therapies, treatment is unsuccessful in 10–30% of cases [18]. Possible causes of unsuccessful treatment are premature termination of treatment, influence of other drugs on therapy, high acidity in the stomach, patient’s health condition, and non-compliance with medical recommendations, but the main cause is antibiotic resistance [18].

Over the past two decades, there has been a significant increase in *H. pylori* resistance to many antibiotics, which are currently the only form of therapy used to treat the infection. The World Health Organization (WHO) places *H. pylori* on the list of pathogens that pose a threat to human health due to the developed antibiotic resistance and require the production of a new antibiotic [4]. The solution to the global problem can be achieved through the discovery of a new antibiotic or an effective vaccine and further research into the emergence and spread of resistance.

In light of this, and given the confirmed primary CAM and LVX resistance in patients infected with *H. pylori* in southern Croatia (CAM-R 37.5% and LVX-R 5%) [21], we performed molecular genetic analysis of three target genes (*23S rRNA*, *gyrA*, and *gyrB*) by PCR and sequencing, along with computational molecular docking analysis. 

The most frequently observed mutations in CAM-resistant *H. pylori* isolates are located at positions 2142 and 2143, where a transition from adenine to guanine (A2142G and A2143G) and a conversion from adenine to cytosine (A2142C) occur. The mutations mentioned are located in domain V of the *23S rRNA* gene. Many researchers consider these point mutations to be the main cause of resistance to CAM. Versalovic and coworkers found for the first time that an A-to-G transition mutation at a position matching the *23S rRNA* position 2058 and 2059 of *E. coli* is associated with *H. pylori* resistance to CAM and the failure of eradication [19]. The mutation conferring resistance to macrolides (such as CAM) occurred at or near the methylation site (cognate with position 2058 of the *23S rRNA* of *E. coli*) of the 23S rRNA of *Bacillus stearothermophilus* [22], which was previously known to confer macrolide resistance.

In 2021, Albasha M.A. et al. conducted a study in Sudan to determine the resistance of *H. pylori* to CAM [23]. By sequencing the PCR products, they detected A2142G and A2143G mutations, which are consistent with the A2142G and A2143G mutations found in this study. Marques T.A. et al. (2020), in a study conducted in Portugal, used DNA samples isolated from biopsies of gastric mucosa and detected mutations associated with CAM-resistance using the next-generation sequencing (NGS) method [24]. They found the A2142C mutation in 16.7% of the isolates.

Additional mutations were also observed at positions 2182 and 2244 (T2182C and T2244C). They occurred most frequently together with mutations at positions 2142 and 2143. Marques T.A. et al. (2020) considered that the T2182C mutation was not associated with CAM-resistance because it was found in susceptible strains [24]. The opposite opinion was expressed by Kim. S.K. et al. (2002) and Khan R. et al. (2004) in their works confirming the association between the T2182C mutation and resistance to CAM [25,26]. Kim. S.K. et al. (2002) in South Korea mapped the T2182C mutation in resistant strains using the PCR-RFLP method [26]. Similarly, Khan R. et al. (2004) in Bangladesh detected the presence of the T2182C mutation in resistant strains using the PCR method and sequencing the PCR products [25]. The results of their work are consistent with the results of this study, as T2182C was detected in 71.43% of resistant strains. Recent evidence suggests a relationship between the type and number of point mutations and the degree of CAM- resistance. Binkowska et al. found that isolates with multiple mutations in the *23S rRNA* gene, particularly at different positions, tended to have a higher resistance [27]. The cumulative effect of multiple mutations may lead to a synergistic reduction in CAM binding, making treatment less effective. The degree of resistance or susceptibility of *H. pylori* to CAM is closely related to the specific point mutations in the *23S rRNA* gene. Mutations at positions 2142 and 2143, including A2142G and A2143G, are particularly associated with high-level resistance to CAM [27]. These mutations are located in domain V of the *23S rRNA* gene, which is a primary binding site for macrolide antibiotics [28]. These changes interfere with the binding of CAM to the ribosome, making the antibiotic less effective at inhibiting protein synthesis. Consequently, *H. pylori* strains carrying these mutations show a reduced sensitivity to CAM, leading to treatment failure. The T2182C [27] and T2244C [29] mutations have also been associated with resistance to CAM. Although these mutations do not confer as high a level of resistance as the A2142G or A2143G mutations, they still interfere to some extent with the binding of CAM to the ribosome. As a result, strains carrying these mutations may exhibit decreased CAM susceptibility [27,29].

In addition, the effect of the T2244C mutation on the development of resistance has not been fully investigated, but it was found in all resistant strains and in both susceptible strains. Susceptibility testing of *H. pylori* strains to CAM revealed that the isolates HP9 and HP14 were susceptible to CAM, but bioinformatic analysis revealed mutations T2182C and T2244C in the isolate HP9 and mutation T2244C in the isolate HP14, respectively (Table 5). 

The presence of these mutations in phenotypically susceptible isolates raises interesting questions about the evolution of CAM-resistant genotypes. These seemingly contradictory results suggest a dynamic interplay of bacterial genetics, environmental factors, and treatment history. The resistance of *H. pylori* to clarithromycin may develop rapidly in response to antibiotic treatment but may revert to sensitivity without treatment. *H. pylori* isolates from four patients who were sensitive to CAM before antibiotic treatment developed resistance after treatment, suggesting that the bacteria developed resistance to CAM in response to treatment [19]. Interestingly, susceptible isolates were still found in certain patients 4 and 7 months after treatment [19]. This suggests that the resistant mutants may have less of a competitive advantage over wild-type *H. pylori* strains in the absence of therapeutic pressure (i.e., when the antibiotic is no longer present). Consequently, wild-type *H. pylori* strains may displace the resistant mutants, leading to a return to susceptibility. The DNA fingerprints of serial isolates from the same patient that were initially susceptible and later became resistant to clarithromycin were similar [19]. This suggests that the resistant isolates likely originated from the same bacterial clone and were not due to new infections [19]. Certain bacteria in the population carried spontaneous mutations that resulted in resistance to CAM. Point mutations could lie dormant in the population as a genetic reservoir until a specific selection pressure triggers their expression. These mutations may give the bacterium a subtle advantage in certain microenvironments. While they do not necessarily lead to direct resistance, they can alter ribosomal structure and affect the binding of macrolides such as CAM. These changes could lead to phenotypic flexibility that allows the bacterium to survive in the presence of the antibiotic without significant growth inhibition. The development of CAM-resistant genotypes is likely to be gradual. Over time, additional mutations may accumulate that further attenuate the inhibitory effect of CAM and lead to clinically visible resistance.

Docking analysis was performed to investigate the influence of these mutations on the binding of CAM. Zhang et al. resolved the cryo-EM structure of the *Mycobacterium tuberculosis* 50S ribosome subunit bound with CAM and analyzed the interactions [30]. They demonstrated that CAM binds at the nascent polypeptide exit tunnel (NPET) near the peptidyl transferase center (PTC) which includes the bases: A2059, A2062, A2503, and G2505. This is in agreement with our docking analysis on the wild type 23S rRNA subunit from *Mycobacterium tuberculosis*, where the indicated nucleic bases correspond to A2142 and A2146 from *H. pylori*, while A2503 and G2505 are in the region of the 23S rRNA subunit that does not have a similar sequence (in *M. tuberculosis* and *H. pylori*) and they do not have a match in *H. pylori*. This indicates that the mutations of these bases will influence CAM binding. On the other hand, the mutations T2182C and T2244C are spatially separated from the CAM binding site (see Figure S1) and may have less effect on binding strength. It is possible that these mutations influence the overall structure of 23S rRNA and in such a way alter CAM binding. But, as the docking gives a static picture of a protein and it will not give us change in an overall structure of a 23S rRNA, we proceeded to investigate the docking of CAM to A2142G, A2142C, and A2143G mutants. 

The FingeRNAt tool identified interactions with the A2300 nucleic base, the so-called “gate site” (corresponding to A2062 in the *M. tuberculosis*), which is one of the key residues to interact with CAM and is involved into protein translation control [31]. Other interaction partners of CAM in all variants include the nucleic bases: A2142, A2146, C2669, G2691, C2693, and C2694 where interactions are either hydrogen bonds or lipophilic interactions. Additionally, mutants include interactions with C1864 and T2692. We analyzed clashes to investigate destabilizing interactions, and found three clashes in the mutants A2296G and A2297G (with nucleic bases A2400, G2141, and one in the nonoverlapped region) and one in A2296C. These clashes are not present in the wild type structure (pdb:7F0D) and induce destabilization in the interactions of mutants with CAM. The importance of the A2143G mutation is also pointed out in the Chu et.al. research [28].

Levofloxacin is a fluoroquinolone antibiotic that targets bacterial DNA gyrase, an important enzyme involved in DNA replication and transcription [32]. DNA gyrase consists of two subunits, A and B, encoded by the *gyrA* and *gyrB* genes, respectively [33]. Point mutations in these genes can lead to resistance to LVX. By analyzing the amino acid sequences of subunit A (GyrA) from LVX-resistant *H. pylori* strains, we detected point mutations in the QRDR region at position 91 (D91G and D91N). The QRDR is a region within the DNA gyrase enzyme that interacts directly with quinolone antibiotics [32]. These mutations impair the binding of LVX to the enzyme and reduce the inhibitory effect of the drug [32]. Previous research by various groups has consistently linked these mutations to reduced susceptibility to LVX. In 2020, Zhang Y. et al. in China detected a mutation at position 91 (D91N) in LVX-resistant *H. pylori* strains isolated from the gastric mucosa of children [34]. In the same year, Lok et al. in Beijing [35] identified the D91G mutation in *H. pylori* isolated from gastric mucosa by the PCR method and sequencing, which was also found in this study. The substitution of the amino acid asparagine (N) at position 87 in the QRDR region is also responsible for resistance to LVX [36], but it was not detected in the isolates of this study. 

When we look at the docking of LEV to GyrA, we can see the difference in the binding between wild type and mutants. In the case of the D91G mutation, the negatively charged Asp91 amino acid is exchanged for the smallest amino acid glycine which has only hydrogen in the side chain. This change influences the interactions of LVX with GyrA (compare the right panel of Figure 3): there are less polar and Van der Waals (VdW) interactions and more VdW clashes. This leads to less stable interactions. In contrast, for the D91N mutation, the negatively charged Asp91 is exchanged for polar uncharged amino acid asparagine. This caused a different orientation of the carboxyl group of LVX, which no longer has a negatively charged carboxyl group from D91 in the GyrA. Changes in the interactions are shown in the right part of Figure 3 which are not pronounced in the docking study and the molecular dynamics would be needed to investigate the influence of D91N. This is outside the scope of the present work and indicates that further study is needed to understand the D91N mutant’s resistance to LVX. But, the importance of this residue is also highlighted in the Chu et. al. paper, performed on the *E. coli* strand of *H. pylori* [28], with the additional mutation of N87K.

Upon analysis of the amino acid sequences of subunit B (GyrB) from LVX-resistant *H. pylori* strains, we found point mutations at positions N481E, N481D, and R484K. These mutations are located near the interface between the GyrA and GyrB subunits, suggesting that they may indirectly affect LVX binding. The specific mechanisms by which these GyrB mutations contribute to resistance are not fully understood. However, it is suspected that they may affect the conformation and function of the DNA gyrase enzyme and ultimately reduce LVX binding and its inhibitory activity [32]. The isolate HP19 exhibits resistance to LVX, although it does not contain mutations in GyrA, but it does contain the mutations N481E and R484K in GyrB (Table 5). For all these reasons, there is a possibility that the N481E and R484K mutations are associated with resistance to LVX.

For the atomic analysis of GyrB, we performed docking for the wild type and the single point mutant N481D, and two simultaneous mutations N481D&R484K and N481E&R484K. In the N481D mutant, the polar uncharged amino acid asparagine (N) was substituted with negatively charged aspartic acid (D). This change caused the loss of VdW interactions and induced more VdW clashes which can destabilize LVX binding. In the two mutants with two simultaneous mutations, the positively charged amino acid Arg484 was exchanged by another positively charged amino acid lysine, while the polar uncharged amino acid asparagine was replaced by the negatively charged aspartic acid (D) or glutamic acid (E). Comparisons of the docked pose of LVX in the wild type and N481E&R484K are shown in the left panel of Figure 4 and show the loss of the ionic interactions (dotted green lines) between R484 and LVX in the N481E&R484K mutant. Additionally, the number of polar interactions decreased which all led to the destabilization of interactions.

Overall, molecular docking is a valuable tool for predicting ligand–receptor interactions through computational modeling, involving the optimization of ligand orientation and the evaluation of binding energies using a scoring function. The activity of the proteins is based on their possibility to change their structure, and this is hard to model within docking studies and exceeds the scope of the present contribution. Additional studies are needed to explore the role of protein flexibility on the interaction of GyrA and GyrB with LVX and 23S rRNA and CAM. 

The reciprocal relationship between mutations in the *gyrA* and *gyrB* genes is an important aspect of LVX resistance. Mutations in either subunit of DNA gyrase can lead to resistance, but the presence of mutations in both subunits often leads to a higher level of resistance. This is due to the fact that the two subunits work together to form the functional DNA gyrase enzyme complex [32]. Mutations in one subunit can compensate for the decreased drug binding caused by mutations in the other subunit, resulting in a decreased sensitivity to LVX. The relationship between specific point mutations and the degree of resistance or susceptibility to LVX may vary. Different mutations may have different effects on the binding of LVX and thus on the overall degree of resistance. In addition, the combination of mutations in GyrA and GyrB may synergistically contribute to higher levels of resistance.

**Figure 5:**
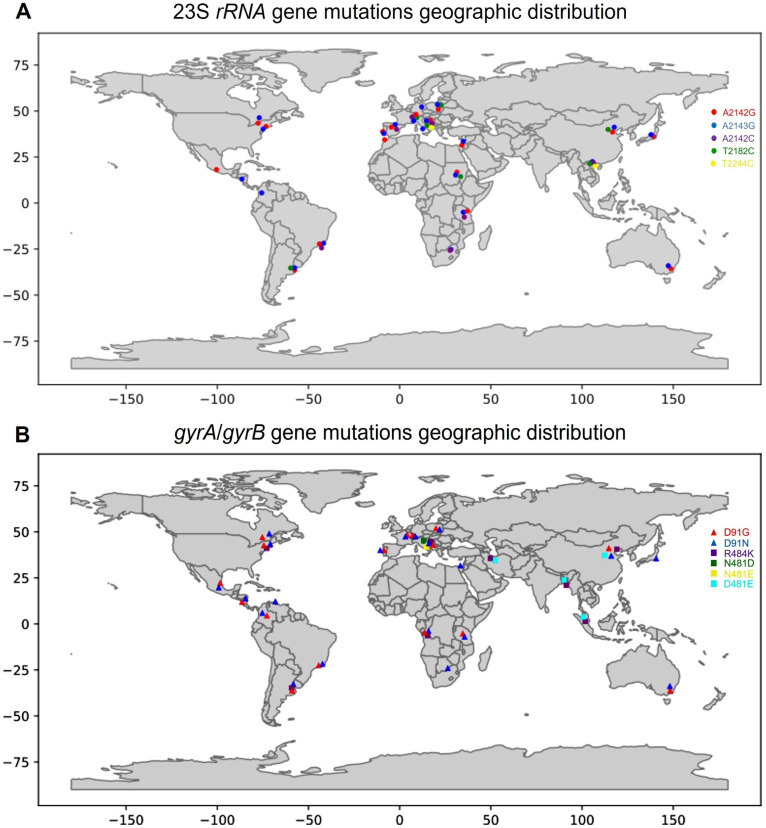
The presence and diversity of mutation. Global distribution of key mutations in *23S rRNA* (**A**), and *gyrA* and *gyrB* (**B**) *H. pylori* genes by country. Python 3.9 has been used to process the input data. The data were loaded from ‘csv’ using the ‘pandas’ library, and geographic markers were stored in tuple-in-a-dictionary format. After preprocessing, the visualization of gene mutation and antibiotic resistance on a world map was performed using the ‘geopandas’ and ‘matplotlib’ libraries.

To comprehend the global distribution of mutations associated with CAM and LVX resistance, a snowball search approach was used (Figure 5). This approach involves an iterative process of data collection from references and citations in existing scientific articles [19,20,23,24,27,29,34,35,36,37,38,39,40,41,42,43,44,45,46,47,48,49,50,51,52,53]. The primary keywords related to point mutations in the *23S rRNA*, *gyrA*, and *gyrB* genes of clarithromycin- and levofloxacin-resistant *H. pylori* isolates served as the starting point for the search.

The global distribution of key mutations in the *23S rRNA*, *gyrA*, and *gyrB H. pylori* genes (Figure 5) is not restricted to a particular population or strain of *H. pylori*, and their distribution is likely influenced by factors such as antibiotic use, geographic location, and other genetic and environmental factors.

Knowledge of resistance-related mutations in the *23S rRNA*, *gyrA*, and *gyrB* genes and associated minimum inhibitory concentration (MIC) susceptibility profiles is critical for tailored, effective therapy against *H. pylori*, especially considering regional differences, as it allows clinicians to make informed decisions to maximize treatment efficacy and minimize the risk of treatment failure due to antibiotic resistance. The type of point mutations in the *23S rRNA* gene can differentially affect the MIC values of CAM, reflecting the degree of resistance [54]. Versalovic suggested the MIC value of 32 as a cut-off point between low and high antibiotic resistance [54]. In cases where high-level resistance mutations (A2143G) are detected [27], alternative antibiotics should be considered a priori to improve treatment efficacy. Conversely, strains with lower MIC values due to mutations such as T2182C (MIC ≤ 32) [27] still require a cautious approach to clarithromycin-based therapy.

During the studies, dual resistance was detected, i.e., simultaneous resistance to CAM, a macrolide antibiotic, and LVX, a fluoroquinolone antibiotic, which belong to different antibiotic groups. According to the data in Table 5, the isolates HP2, HP5, HP8, and HP19 exhibit resistance to both antibiotics, which is consistent with the mutations detected in the sequences of the three genes. Similar data were provided by [35], who detected simultaneous resistance to CAM and LVX in 22 of a total of 112 samples [35]. Multiple resistance to both antibiotics is an obstacle in treating the infection, as CAM is part of the “first line of defense” and LVX is among the last forms of alternative therapies [7], which significantly reduces the number of possible therapies. As options for effective treatment diminish, clinicians may resort to antibiotics they previously avoided out of concern for resistance development. This may inadvertently promote the emergence of strains that exhibit a broader spectrum of resistance, further compromising treatment outcomes.

Our preliminary molecular analysis targeted point mutations in genes associated with resistance to CAM and LVX. Although limited in scope, this analysis provided valuable insights into the prevalence of resistance-conferring mutations in southern Croatia. The identified mutations in the *23S rRNA*, *gyrA*, and *gyrB* genes have potential clinical implications and suggest the possible development of treatment strategies tailored to individual resistance profiles. Beyond our preliminary findings, a comprehensive research approach that includes a larger cohort of patients and employs multiple methods is of great importance. Incorporating patient demographics, clinical symptoms, previous treatments, and MLST analyses of *H. pylori* strains would provide a holistic understanding of antibiotic resistance patterns.

This comprehensive approach serves several important purposes:

1. Identification of primary and secondary resistance: Distinguishing between primary and secondary resistance is critical for making informed treatment decisions. Comprehensive patient data could provide insight into whether resistance arose from previous antibiotic exposure or from intrinsic genetic factors.
2. Population genotyping and resistance correlation: MLST analysis would elucidate the genetic diversity of local *H. pylori* strains. Linking this diversity to resistance patterns could provide insight into population dynamics that influence the evolution of antibiotic resistance.
3. Treatment optimization: A more nuanced understanding of patient demographics, clinical symptoms, and resistance mechanisms would enable the development of personalized treatment regimens and an increase in treatment success rates.
4. Epidemiologic insights: Comprehensive research would contribute valuable data to the global understanding of *H. pylori* antibiotic resistance, supporting the formulation of effective public health strategies.

## 4. Materials and Methods

### 4.1. Isolation of H. pylori

*H. pylori* strains for antibiotic susceptibility testing were cultured from biopsy specimens of gastric mucosa on Pylori agar (bioMerieux, Marcy l’Etoile, France) after incubation for 3–5 days at 37 °C in a microaerophilic atmosphere. Susceptibility testing of *H. pylori* isolates to amoxicillin, CAM, tetracycline, LVX, and metronidazole was determined by E-test (AB Biodisk, Solna, Sweden). E-tests were performed on Columbia agar plates containing 7% horse blood without supplement. Plates were inoculated with a bacterial suspension (turbidity of 3–4 McFarland) and incubated for 72 h at 37 °C in a microaerophilic atmosphere. Antibiotic cut-off points were determined following the European Committee on Antimicrobial Susceptibility Testing (EUCAST; Clinical Breakpoint Table v. 5.0) criteria: >0.125 mg/L for amoxicillin, >0.5 mg/L for CAM, >1 mg/L for tetracycline, >1mg/L for LVX, and >8 mg/L for metronidazole [55]. Gastric tissue samples were collected during upper endoscopy from patients diagnosed with gastrointestinal disease. Cultivation of *H. pylori* strains was performed at the University Hospital Split in the Department of Clinical Microbiology and Parasitology.

### 4.2. Isolation of Genomic DNA from H. pylori

Colonies of clinical *H. pylori* isolates were scraped from blood agar plates for DNA extraction. Genomic DNA was isolated using the commercial NucleoSpin^®^ Microbial DNA Mini Kit for DNA from microorganisms (MACHEREY-NAGEL GmbH & Co. KG, Düsseldorf, Germany) according to the manufacturer’s instructions. DNA quality was assessed using a spectrophotometer (NanoPhotometer N60 ^®^, IMPLEN, München, Germany).

### 4.3. Amplification of the 23S rRNA, gyrA, and gyrB Genes

The PCR method was used for amplification of domain V of the *23S rRNA*, *gyrA*, and *gyrB* genes. The PCR reaction conditions and the nucleotide sequence of the primers used for the amplification of the target genes are listed in Table 6.

Based on the concentrations and purity of the isolated DNA samples, three PCR reaction mixtures were prepared for amplification of the *23S rRNA*, *gyrA*, and *gyrB* genes (1, 2, and 5 µL of DNA template were added to 30 µL of reaction mixture, giving a total of 25 ng/μL of DNA template per sample). The ingredients for the PCR reaction mixture were added to the previously labelled tubes according to Table 6.

Visualization and confirmation of the amplicon fragments was performed by DNA electrophoresis on a 1% (*w*/*v*) agarose gel. Fragments were purified using the Wizard SV Gel and PCR Clean-Up System Kit (Promega, Madison, WI, USA) according to the manufacturer’s instructions.

### 4.4. Sequencing and Identification of the Mutation

The amplified fragments of the *23S rRNA*, *gyrA*, and *gyrB* genes were sequenced in two directions (forward and reverse) using the Sanger sequencing method at the biotechnology company Macrogen Europe (Amsterdam, The Netherlands).

The sequences of domain V of the *23S rRNA* gene, *gyrA*, and *gyrB* from nine *H. pylori* isolates were multiple aligned and manually edited using the ClustalX tool of the BioEdit program (interface) [59,60]. Visualization and further processing of the aligned sequences was performed using the Jalview program [61].

### 4.5. Molecular Docking Analysis

Molecular docking is a computational method for predicting the preferred mode of binding and affinity between a small molecule (ligand) and a target protein (receptor). It is widely used in drug discovery and in the study of protein–ligand interactions.

Docking simulations were carried out to inspect interactions of the studied antibiotics (LVX and CAM) on the proteins GyrA and GyrB and the 23S rRNA ribosome subunit. 

Key components of docking include the optimization algorithm and the scoring function. The optimization algorithm identifies rotatable bonds in the ligand’s structure and adjusts its orientation within the docking site. Meanwhile, the scoring function assesses the quasi-energy of the resulting receptor–ligand complex. The binding score is iteratively evaluated during optimization to identify the structure with the lowest energy.

The number of active torsions, or rotatable bonds, in the ligand’s structure affects the complexity of the docking process. A higher number of potential rotations makes finding the optimal binding position more challenging.

In the AutoDock Vina software v1.2.3 [62] used in this research, the scoring function combines intermolecular and intramolecular contributions to calculate the binding free energy. The optimization process employs the iterated local search global optimizer algorithm. On the other hand, for the 23S rRNA–ligand docking HADDOCK v2.4-2022.08 [63,64] was used. HADDOCK’s ability to incorporate experimental restraints and flexibility makes it a powerful tool for predicting DNA–ligand interactions.

#### 4.5.1. GyrA and GyrB with LVX

The structure of the antibiotic LVX was taken from PubChem data base [65] (CID: 149096). AlphaFold DB, developed by DeepMind, is an advanced protein structure prediction model utilizing deep learning. Designed for accurate protein folding, it employs a specialized deep neural network architecture. By analyzing a vast dataset of known protein structures and sequences, it learns the intricate connections between amino acid sequences and their 3D structures. The model’s prediction process involves two key steps: firstly, it forecasts distances between amino acid pairs, indicating spatial relationships; subsequently, it calculates protein backbone and side chain orientations based on these distances. This information contributes to generating a projected 3D protein structure. AlphaFold DB’s applicability spans diverse organisms, encompassing humans, animals, and microorganisms. Notably, the model achieves exceptional predictive precision, often rivaling or outperforming experimental methods like X-ray crystallography and cryo-electron microscopy.

AutoDock Vina software v1.2.3 was used for the protein–antibiotic docking. The preparation of the ligands for the docking was performed using the Chimera [42] Prep Dock Tool, which calculates charges for the ligand using the AM1-BCC method. Docking was performed with the default parameters, including energy_range = 3; exhaustiveness = 8; num_modes = 10. Docking was performed with flexible ligands to rigid GyrA within the docking box, position (−10, 15, −7), size (25, 25, 25); and to GyrB, position (17, −20, −20), size (25, 25, 25). The Pymol [URL citation: The PyMOL Molecular Graphics System, Version 1.2r3pre, Schrödinger, LLC.] Mutagenesis Tool was used to induce mutations into the GyrA structure (D91G and D91N). Interatomic interactions were analyzed using the Arpeggio web server for calculating and classifying interactions in 15 different categories based on atom type, distance, and angle terms [66]. Chimera [67] and Pymol software PyMOL 2.5.0 facilitated the visual examination and depiction of different complexes, along with the creation of graphical representations.

#### 4.5.2. 23S rRNA and CAM

The structure of CAM was taken from the PubChem data base (CID: 84029). A search for the structure of 23S rRNA from *H. pylori* (Genbank: U27270.1) did not result in the crystal structure from *H. pylori* but those from different organisms. Among these was the cryo-EM structure of the *Mycobacterium tuberculosis* 50S ribosome subunit bound with CAM [30] (PDB: 7F0D). Its sequence similarity for isolated 23S rRNA is 58%, and the mutated positions we are interested in have the same nucleic base. Taking this into account, we performed docking studies on this structure. Mutated positions in *H. pylori* include: A2142, A2143, T2182, and T2244, and correspond to: A2296, A2297, T2335, and T2397 in the 23S rRNA ribosome subunit from *Mycobacterium tuberculosis* (PDB: 7F0D). A visual inspection of the 23S rRNA with CAM (PDB: 7F0D) revealed that it interacts with A2296 and A2297, which allowed us to focus on mutation in this position (compare with Figure S1). For this reason, the mutations A2296G, A2296C, and A2297G were induced using the Pymol Mutagenesis Tool and submitted to the HADDOCK v2.4-2022.08 web server. Docking was performed using default parameters for DNA molecules, with interaction partners on 23S rRNA indicated as A2296, A2297, C2848, and U2849. The results were clustered and the first pose in Cluster “1” was selected for interaction analysis which was carried out using FingeRNAt [68]. FingeRNAt serves as a Python 3.10.12 software utility designed to identify non-covalent interactions that arise within nucleic acid–ligand complexes. Visualization and graphical representations were performed using Chimera [67].

## 5. Conclusions

Our preliminary investigation on *H. pylori* antibiotic resistance is a first step toward a deeper understanding of this issue. A comprehensive research approach would provide insights into the complex interplay of patient characteristics, bacterial genetics, and antibiotic resistance at the atomic level. Molecular docking analysis showed that point mutations alter the binding interactions between targets and antibiotics. Extensive study including molecular dynamics could shed light on the dynamical properties of the target/antibiotic complex and provide atomic explanations of the observed resistance. Ultimately, such all-encompassing research could revolutionize the treatment of *H. pylori* infections by supporting the development of targeted therapies and providing information for public health policy to curb the rise of antibiotic-resistant strains.

## Figures and Tables

**Table 1:** Comparison of antibiogram results with mutation analysis of the V domain of the *23s rRNA* gene.

Isolate	Sensitivity to CAM	Mutation Site
HP2	resistant	A2143G, T2182C, T2244C
HP5	resistant	A2143G, T2244C
HP8	resistant	A2142G, T2182C, T2244C
HP9	sensitive	T2182C, T2244C
HP10	resistant	A2142G, T2182C, T2244C
HP11	resistant	A2143G, T2182C, T2244C
HP14	sensitive	T2244C
HP19	resistant	A2142G, T2182C, T2244C
HP20	resistant	A2142C, T2244C

**Table 2:** *gyrA* gene mutations and amino acid changes in LVX-resistant *H. pylori* isolates.

Isolate	Base Mutation	Amino Acid Change	Point Mutation
HP5, HP11	AAC → AAT	-	-
HP5, HP8	GAT → AAT	D → N	D91N
HP2	GAT → GGT	D → G	D91G

**Table 3:** GyrB gene mutations and amino acid changes in *H. pylori* isolates.

Isolate	Base Mutation	Amino Acid Change	Point Mutation
HP2, HP5, HP8, HP9,	**A**A**T** → **G**A**G**	N → E	N481E
HP10, HP11, HP14	**A**AT → **G**AT	N → D	N481D
HP2, HP5, HP8, HP9, HP11	A**G**A → A**A**A	R → K	R484K

**Table 4:** Comparison of antibiogram results with the mutation analysis of subunits A (GyrA) and B (GyrB) of DNA gyrase.

Isolate	Susceptible to LVX	Point Mutation in Subunit A of DNA Gyrase	Point Mutation in Subunit B of DNA Gyrase
HP2	resistant	D91G	N481E, R484K
HP5	resistant	D91N	N481E, R484K
HP8	resistant	D91N	N481E, R484K
HP9	sensitive	no mutation	N481E, R484K
HP10	sensitive	no mutation	N481D
HP11	sensitive	no mutation	N481D, R484K
HP14	no data	no mutation	N481D

**Table 5:** Susceptibility to CAM and LVX and resistance-related mutations in *H. pylori* isolates.

Isolate	Susceptibility to CAM	Mutation Site in *23s rRNA* Gene	Susceptibility to LVX	Point Mutation in GyrA	Point Mutation in GyrB
HP2	resistant	A2143G, T2182C, T2244C	resistant	D91G	N481E, R484K
HP5	resistant	A2143G, T2244C	resistant	D91N	N481E, R484K
HP8	resistant	A2142G, T2182C, T2244C	resistant	D91N	N481E, R484K
HP9	sensitive	T2182C, T2244C	sensitive	no mutation	N481E, R484K
HP10	resistant	A2142G, T2182C, T2244C	sensitive	no mutation	N481D
HP11	resistant	A2143G, T2182C, T2244C	sensitive	no mutation	N481D, R484K
HP14	sensitive	T2244C	no data	no mutation	N481D
HP19	resistant	A2142G, T2182C, T2244C	resistant	no mutation	N481E, R484K
HP20	resistant	A2142C, T2244C	sensitive	no mutation	no mutation

**Table 6:** Primers and polymerase chain reaction setup for amplification of the *23S rRNA*, *gyrA,* and *gyrB* genes of *H. pylori*.

Gene	Primer	Primer Sequence (5′→3′)	PCR Conditions	Reaction Mixture (30 µL) per Sample	Amplicon Size (bp)	Reference
*23S rRNA*	Hp23S 1942F Hp23S 2308R	AGGATGCGTCAGTCGCAAGAT CCTGTGGATAACACAGGCCAGT	initial denaturation at 95 °C for 2 min, followed by 5 cycles: 94 °C for 30 s, 60 °C for 30 s, and 72 °C for 30 s; then 30 cycles: 94 °C for 15 s, 60 °C for 15 s, and 72 °C for 20 s, with a final extension at 72 °C for 7 min	3 µL PCR buffer (10×), 0.3 µL bovine serum albumin (10 μg/μL), 3 µL dNTPs (2 mM), 1.5 µL each of forward (10 µM) and reverse primers (10 µM), 25 ng/μL of DNA template, and 0.3 µL Taq polymerase (5 U/µL) *	367	[56,57]
*gyrA*	gyr APF gyr APR	AGCTTATTCCATGAGCGTGA TCAGGCCCTTTGACAAATTC	initial denaturation 95 °C, 5 min; 35 cycles of amplification: 94 °C, 1 min; 53 °C, 1 min; 72 °C, 1 min; final extension 72 °C, 10 min	3 µL PCR buffer (10×), 0.3 µL bovine serum albumin (10 μg/μL), 3 µL dNTPs (2 mM), 1.5 µL each of *gyrA* and *gyrB* forward (10 µM) and reverse primers (10 µM), 25 ng/μL of DNA template, and 0.3 µL Taq polymerase (5 U/µL) *	582	[58]
*gyrB*	gyr BPF gyr BPR	CCCTAACGAAGCCAAAATCA GGGCGCAAATAACGATAGAA			465	

* The volume of sterile distilled water (µL) depends on the concentration of isolated DNA.

## Data Availability

The partial *23S rRNA* gene sequences of the nine *H. pylori* isolates were deposited in the GenBank database under the accession numbers OR116141–OR116149. The partial coding sequence (CDS) of *gyrA* and *gyrB* of the seven *H. pylori* isolates were deposited in GenBank under the accession numbers OR147771–OR147784.

## Supplementary Materials

The following supporting information can be downloaded at: https://www.mdpi.com/article/10.3390/ijms241914560/s1.
