# Supplementary material for: Molecular Characterization and Mutational Analysis of Clarithromycin- and Levofloxacin-Resistance Genes in Helicobacter pylori from Gastric Biopsies in Southern Croatia
Source: Int J Mol Sci. 2023 Sep 26;24(19):14560. doi: 10.3390/ijms241914560 (PMC10572715; doi:10.3390/ijms241914560)
Supplement: Supplementary file 1 [file ijms-24-14560-s001.zip › ijms-2614112-supplementary.pdf]

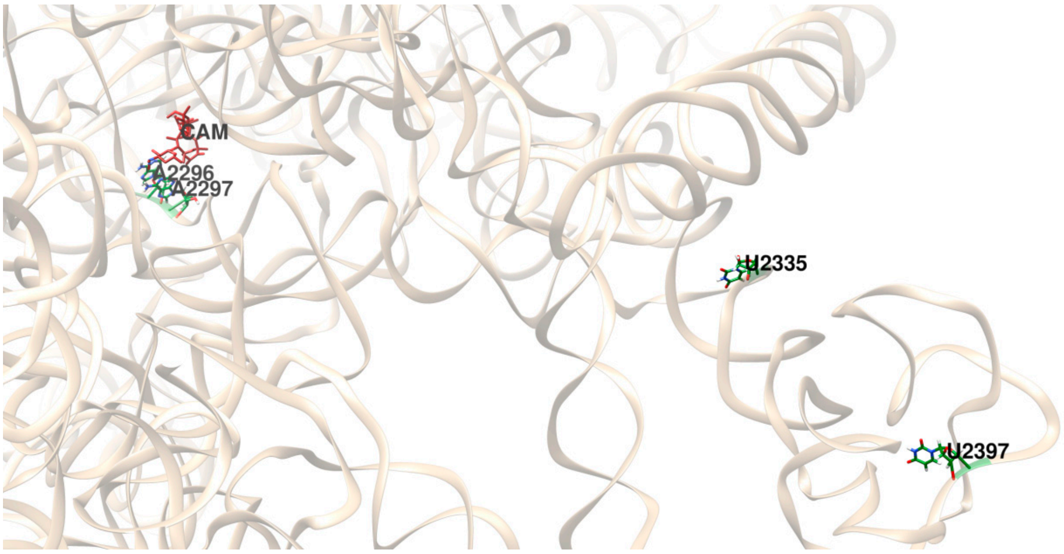

Figure S1. The position of the mutated nucleic basis on the *M. tuberculosis* 23S rRNA subunit. Mutated nucleic basis are shown in the stick representation, colored green, while Clarithromycin (CAM) is colored red. Nucleic basis A2296 and A2297 (corresponding to A2142 and A2143 in *H.pylori*) are in the CAM binding site, while U2335 and U2397 (corresponding to T2182 and T2244 in *H.pylori*) are not.
